# African *Neotermes*: redescriptions of imago and soldier castes of *N. aburiensis* and *N. agilis* (Isoptera, Kalotermitidae)

**DOI:** 10.3897/zookeys.683.13064

**Published:** 2017-07-06

**Authors:** Ebenezer O. Onagbola, Rudolf H. Scheffrahn

**Affiliations:** 1 Department of Biology, Federal University of Technology, P. M. B. 704, Akure, Ondo State, Nigeria; 2 Fort Lauderdale Research and Education Center, University of Florida, 3205 College Avenue Davie, Florida 33314, U.S.A.

**Keywords:** Taxonomy, Ghana, Nigeria, setae, phragmotic, venation

## Abstract

We redescribe the winged imagos and soldiers of two equatorial African species, *Neotermes aburiensis* Sjöstedt and *N. agilis* (Sjöstedt) and describe their unique character attributes. The imago of *N. aburiensis* is adorned with unique spatulate-pointed setae. The soldier of *N. aburiensis* is unique among *Neotermes* in possessing a phragmotic forehead. The imago of *N. agilis* is small and unique among *Neotermes* in having complete coalescence of radial sector and median veins in the forewing while the soldier of *N. agilis* has a broad pronotum.

## Introduction

*Neotermes* Holmgren consists of 117 extant species (Krishna et al. 2013) making it the second largest kalotermitid genus after *Glyptotermes* Froggatt. Of these, 48 species are described from the soldier or the imago only and 17 (16 from soldier only) are described from mainland China (Krishna et al. 2013). Among the Kalotermitidae, *Neotermes* are usually large species. The imagos are recognized by wing venation in which the media is sclerotized and runs close and parallel to the radial sector to the wing tip. Soldiers possess a long head lacking prominent anterolateral prominences and a faintly depressed frons with a slope of less than 45 degrees (Krishna 1961).

The diversity of *Neotermes* from equatorial central and western Africa is limited to nine species: *N. aburiensis*, *N. agilis*, *N. camerunensis* (Sjöstedt), *N. collarti* Coaton, *N. gestri* Silvestri, *N. lepersonneae* Coaton, *N. nigeriensis* (Sjöstedt), *N. pallidicollis* (Sjöstedt), and *N. sepulvillus* (Emerson). We recently acquired specimens of *N. aburiensis* and *N. agilis* both of which, coincidently, transcend the morphological boundaries of *Neotermes* given above. To contribute to a clearer understanding of African *Neotermes*, we redescribe these two species, including a first description of the *N. agilis* soldier, and highlight their unique morphological characters.

## Material and methods

Photographs of imagos and soldiers were taken as multi-layer montages using a Leica M205C stereomicroscope controlled by Leica Application Suite version 3 software. Preserved specimens were taken from 85% ethanol and suspended in a pool of Purell^®^ Hand Sanitizer to position the specimens on a transparent Petri dish background. Wings (Fig. 3) were mounted on slides using PVA medium (BioQuip, Rancho Dominquez, CA). Montage microphotographs (Fig. 2) were taken from PVA slide mounts of pronotum cuticle (lateral margins) using a Leica CTR 5500 compound microscope.

## Taxonomy

### Neotermes aburiensis

Taxon classificationAnimaliaORDOFAMILIA

Sjöstedt, 1925

Neotermes aburiensis Sjöstedt 1925: 39 (soldier described; Ghana).
Neotermes aburiensis : Grassé 1937: 10-14 (imago and soldier described; Ivory Coast).
Neotermes aburiensis : Coaton 1955: 109-113 (soldier described; Congo-Zaire, Ghana, Ivory Coast).

#### Material examined.

NIGERIA, Ijare; 7.36, 5.17; 9 Apr 2016, E. Onagbola, UF no. AFR2965; 15 alates, nymphs, ex: dead cacao tree. Orita Obele, (OSRC Radio Station Yard), Akure; 7.29, 5.16; 29 Apr 2016 AFR2966; 5 soldiers, 13 alates, nymphs, ex: living stump of Quickstic (*Gliricidia sepiun*) tree. Bayduk Road, Akure; 7.301, 5.151, AFR2971; 1 alate, ex: spider web on house fence.

**Figure 1.**
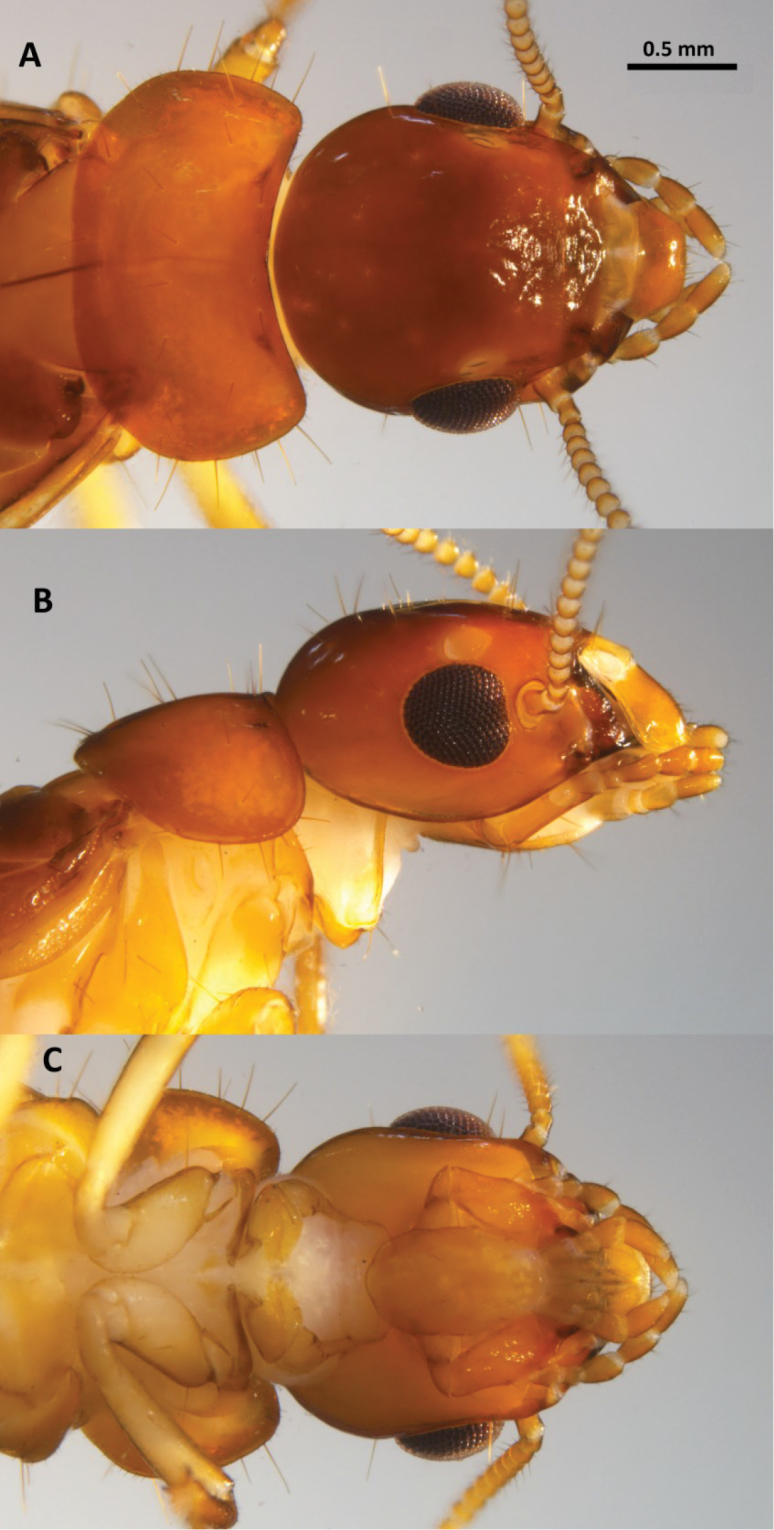
Head and thorax of *Neotermes aburiensis* imago. **A** Dorsal **B** lateral, and **C** ventral views.

**Figure 2.**
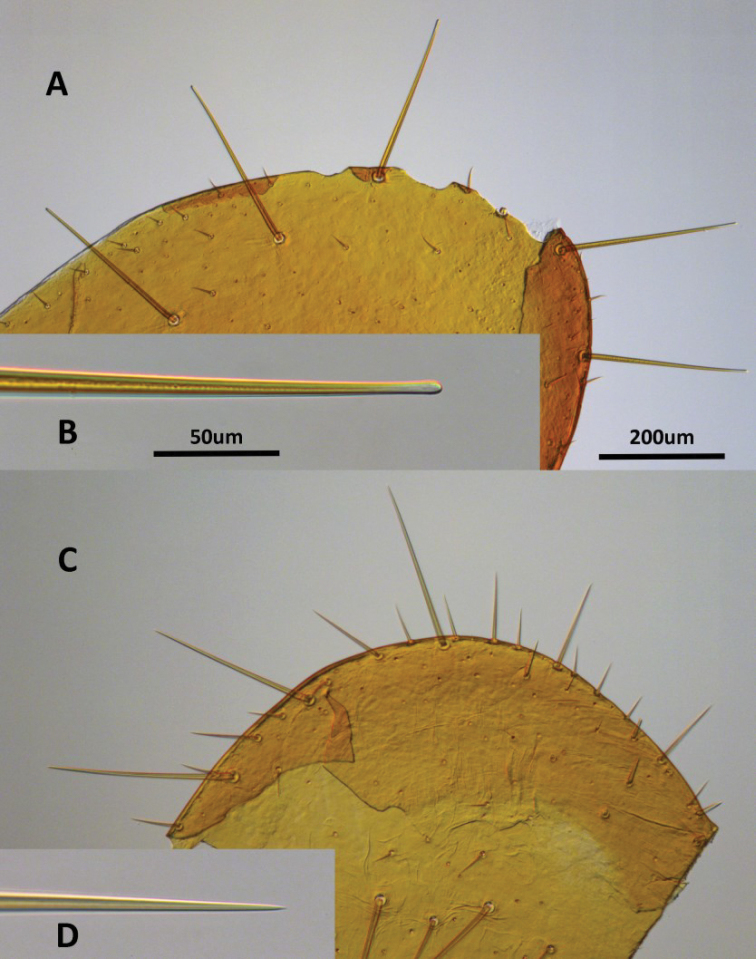
*Neotermes* setae on pronotum cuticle. *Neotermes aburiensis* (**A, B**) and *N. castaneus* (Burmeister) (**C, D**).

**Figure 3.**
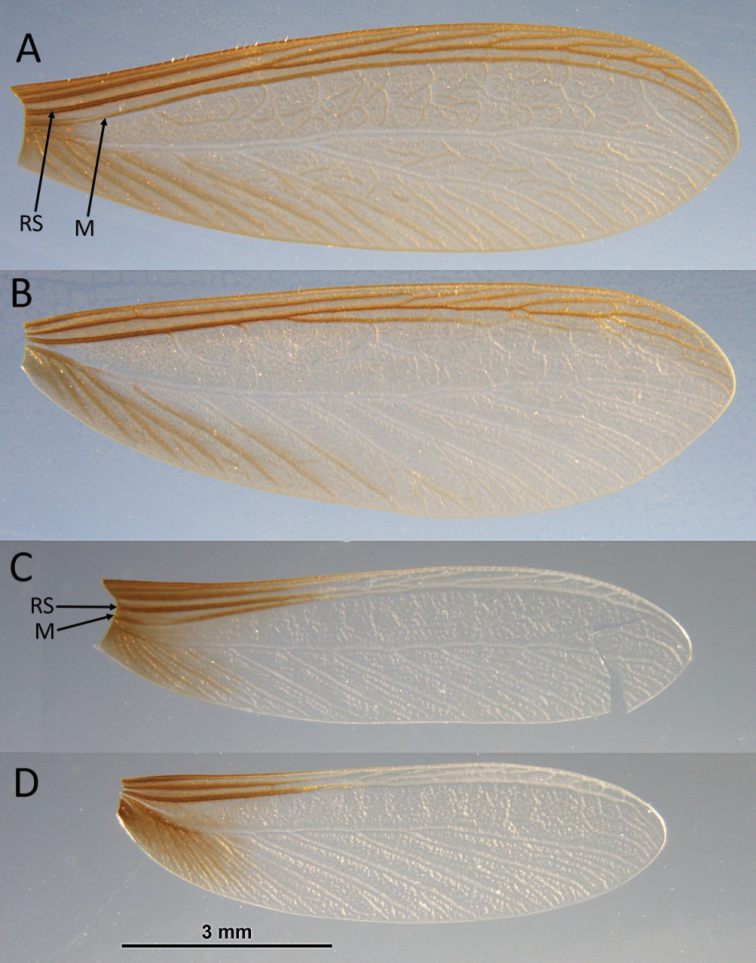
*Neotermes aburiensis* forewing (**A**) and hind wing (**B**). *Neotermes agilis* fore wing (**C**) and hind wing (**D**). RS = radial sector, M = median vein.

*Imago* (Figs 1–3, Table 1). Head with vertex and frons dark reddish-brown grading to light orange-brown at genae. Pronotum lighter than dorsum of head; anteclypeus yellowish and labrum concolorous with genae (Fig. 1). Tergites light yellowish-brown, sternites pale yellow to hyaline. Coxa and femora pale yellow, femora and tarsi contrasting light brown. Chevron pattern from overlapping wing scales and meso and meta notum slightly darker than rest of dorsum. Eyes black; ocelli and antennal articles concolorous with labrum. In dorsal view, lateral margins of head converge to anterior forming distinct trapezoidal outline; cranial sutures absent. Vertex with slight concavity, concavity becoming rugose toward anteclypeus. Eyes of medium size, slightly protruding, and weakly subtriangular. Ocelli large, elliptical, nearly touching eye. Antennae with 17–19 articles, rarely 16. Pronotum in dorsal view collar-shaped. Anterior margin of pronotum very shallowly concave; posterior margin nearly straight; pronotum wider than width of head at eyes; widest at posterior 2/5ths. Scattered long (0.25 mm), erect setae on head and pronotum with numerous short and very short setae (Fig. 2A). Tips of long setae rounded at tip; tip weakly spatulate (Fig. 2B). Costal margin, radius, radial sector, and media of forewing brown and sclerotized along their entire lengths (Fig. 3A, B). Cubitus brown just beyond suture line, with about 10 browish branches to middle of wing 3-4 hyaline branched beyond; membrane between media and cubitus with dozens of faint brown reticulations. Arolia present. Mandible dentition typical of genus; left mandible with posterior margin of first plus second marginal tooth equal to anterior margin of third marginal tooth. Right mandible with posterior margin of second marginal tooth subequal to molar plate.

**Table 1.** Measurements of *Neotermes aburiensis* imago (n=16).

Measurement	max	min	mean
Head length with labrum	2.00	1.83	1.91
Head length to postclypeus	1.58	0.13	0.85
Head width, maximum at eyes	1.68	1.50	1.59
Head height without postmentum	1.05	0.88	0.96
Labrum width, maximum	0.60	0.50	0.55
Eye diameter with sclerite, maximum	0.50	0.43	0.46
Eye to head base, minimum from sclerite	0.28	0.20	0.24
Ocellus diameter, maximum	0.24	0.14	0.19
Ocellus diameter, minimum	0.16	0.10	0.13
Pronotum, maximum length	1.10	0.75	0.93
Pronotum, maximum width	1.93	1.58	1.75
Total length with wings	15.56	13.17	14.37
Total length without wings	8.73	6.19	7.46
Fore wing length from suture	13.17	11.27	12.22
Fore wing, maximum width	3.75	3.00	3.38
Hind tibia length	1.30	0.88	1.09
No. antennal articles	19	16	18

**Figure 4.**
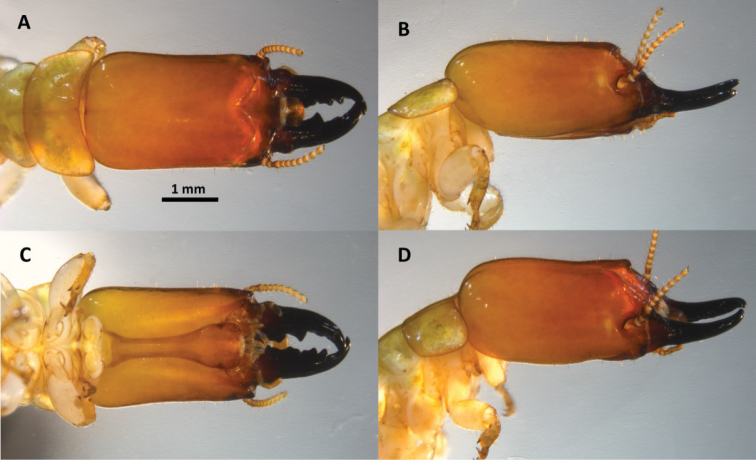
Head and pronotum of *Neotermes aburiensis* soldier**. A** Dorsal **B** lateral **C** ventral, and **D** oblique views.

*Soldier* (Fig. 4, Table 2). Monomorphic. In dorsal view, head capsule dark redish brown at antennae grading to light yellowish orange at occiput. Mandibles black. Three proximal antennal articles reddish brown; distal articles becoming lighter. Eye spots light yellowish brown, elongate, smaller than antennal sockets. Pronotum peripheral margins yellowish orange-brown. Postmentum chestnut; labrum concolorous with vertex. Head capsule in dorsal view, narrowly rectangular; lateral margins slightly concave, narrowing slightly in front. Posterior corners of head evenly rounded; posterior margin rectate. In lateral and oblique view, head capsule almost cylindrical with only slight dorso-ventral compression. In dorsal view, frontal flange squarely angled at 60° forming a deep cleft; in lateral or oblique views antero-lateral corners of flange with knob-like protuberences. Vertex with lateral grooves; lateral margins of vertex with distinct rails. Frons sloping from vertex ≅45°; mandibles curved upward ≅10°. Setae very short and sparse on frons and vertex. Periantennal carina developed; mandibles stout, about one-half length of head capsule; dentition well defined; left mandible with three submarginal teeth; two proximal teeth subdivided by slight concavities; right mandible with proximal tooth eclipsed by labrum; apical teeth angled ≅70°; basal humps moderate, rugose. Labrum linguiform, medium-sized, with long terminal setae. Antennae with 15 articles; third antennal article clavate, barely shorter than fourth and fifth combined. Pronotum broader than head, narrow, shield-shaped; anterior margin evenly concave; anterolateral corners acutely angled at 60°, lateral margins converging to posterior; posterior margin forming an evenly shallow convexity. All femora inflated.

**Table 2.** Measurements of *Neotermes aburiensis* soldier (n=5).

Measurement	max.	min.	mean
Head length to tip of mandibles	5.20	4.30	4.78
Head length to postclypeus	3.90	3.20	3.44
Head width, maximum	2.20	2.10	2.14
Span of frontal processes	1.00	0.88	0.93
Head height, excluding postmentum	1.65	1.50	1.60
Labrum, maximum width	0.53	0.50	0.51
Postclypeus width, maximum	0.60	0.53	0.58
Left mandible length, tip to most distant visible point of ventral condyle	1.70	1.63	1.65
Postmentum, length in middle	2.60	2.30	2.46
Postmentum, maximum width	0.80	0.70	0.77
Postmentum, minimum width	0.38	0.28	0.32
Pronotum, maximum width	2.25	2.08	2.17
Pronotum, maximum length	1.18	1.10	1.13
Hind tibia length	1.30	1.00	1.19
Total length	11.43	10.63	11.02
No. antennal articles	15	15	15

#### Comparisons.

The spatulate-tipped setae on the head and pronotum of *N. aburiensis* are unique among *Neotermes* and possibly all Isoptera which have tappering, needle-like setae (Fig. 2C, D). The clefted frontal flange, its lateral protuberences, and sloping rugose frons form a phragmotic forehead unique among *Neotermes* soldiers.

### Neotermes agilis

Taxon classificationAnimaliaORDOFAMILIA

(Sjöstedt, 1902)

Calotermes agilis Sjöstedt 1902: 302 (imago).
Calotermes agilis Sjöstedt 1904: 15–16 (imago described; Cameroon).
Neotermes agilis , Krishna 1961: 325 (comb. n.).

#### Material examined.

GHANA, Bobiri Butterfly Reserve; 6.690, -1.338, 12 Sept 2006, L.R. Davis, UF no. AFR188; 2 soldiers, 16 alates, nymphs ex: “hard chunk of wood”; 2 alates, 2 nymphs, and soldier photographs sent to K. Krishna 12 Oct 2006.

**Figure 5.**
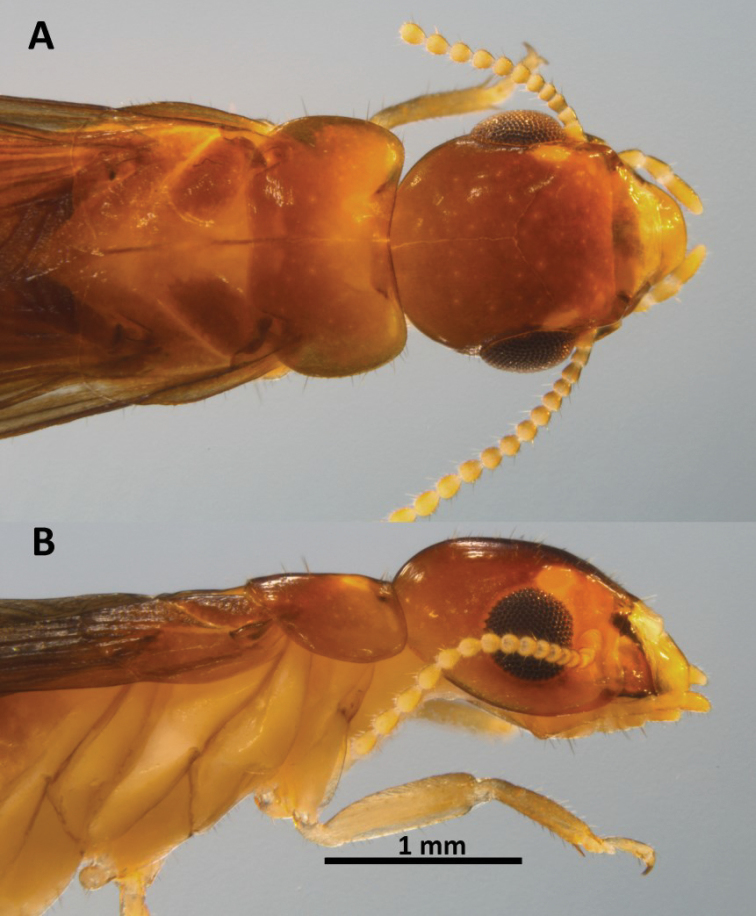
Head and thorax of *Neotermes agilis* imago**. A** Dorsal and **B** lateral views.

*Imago* (Fig. 5, Table 3). Small species. Head with vertex and frons reddish-brown grading to light reddish brown in posterior; lighter near suture of anteclypeus. Pronotum concolorous with dorsum of head except for a lighter area in anterior third; anteclypeus yellowish and labrum concolorous with genae. Tergites yellowish-brown, sternites pale yellow to hyaline. Coxa and femora pale yellow, femora and tarsi light brown. Chevron pattern from overlapping wing scales, meso- and metanotum concolorous with vertex. Eyes greyish black; ocelli and antennal articles concolorous with labrum. In dorsal view, lateral margins of head converge slightely; cranial sutures, thin but distinct. Vertex smooth, slight concavity of frons. Eyes large, slightly protruding, and nearly circular. Ocelli very large, ellipsoid, touching eye. Antennae with 14-16 articles. Pronotum in dorsal view subrectate. Anterior margin of pronotum weakly incised; posterior margin weakly incised in middle; lateral margins evenly rounded. A few scattered erect setae on head; pronotum with numerous long and shorter setae along margins (Fig. 5). Costal margin, radius, radial sector, and media of forewing brown and sclerotized along proximal 1/3 of wing, abrupty becoming hyaline for their remainders (Fig. 3). Median vein with weak posterior bend after suture; coalescing with radial sector at about half length of wing. RS+M hylaline with about 5 anterior branches. Cubitus brown just beyond suture line, with about 4 browish branches to proximal 1/5 of wing 6-7 hyaline branches beyond; membrane pigmented only in basal 1/5; remainder hyaline. Arolia absent. Mandible dentition typical of genus.

**Table 3.** Measurements of *Neotermes agilis* imago (n=10).

Measurement	max	min	mean
Head length with labrum	1.68	1.45	1.54
Head length to postclypeus	1.40	1.13	1.28
Head width, maximum at eyes	1.40	1.15	1.33
Head height without postmentum	0.93	0.75	0.84
Labrum width, maximum	0.58	0.43	0.50
Eye diameter with sclerite, maximum	0.53	0.45	0.49
Eye to head base, minimum from sclerite	0.20	0.13	0.16
Ocellus diameter, maximum	0.22	0.14	0.19
Ocellus diameter, minimum	0.18	0.10	0.14
Pronotum, maximum length	0.85	0.63	0.72
Pronotum, maximum width	1.53	1.30	1.41
Total length with wings	12.70	10.16	11.16
Total length without wings	6.98	5.40	5.98
Fore wing length from suture	10.32	8.25	8.90
Fore wing, maximum width	2.75	1.95	2.24
Hind tibia length	1.00	0.90	0.94
No. antennal articles	16	14	15

**Figure 6.**
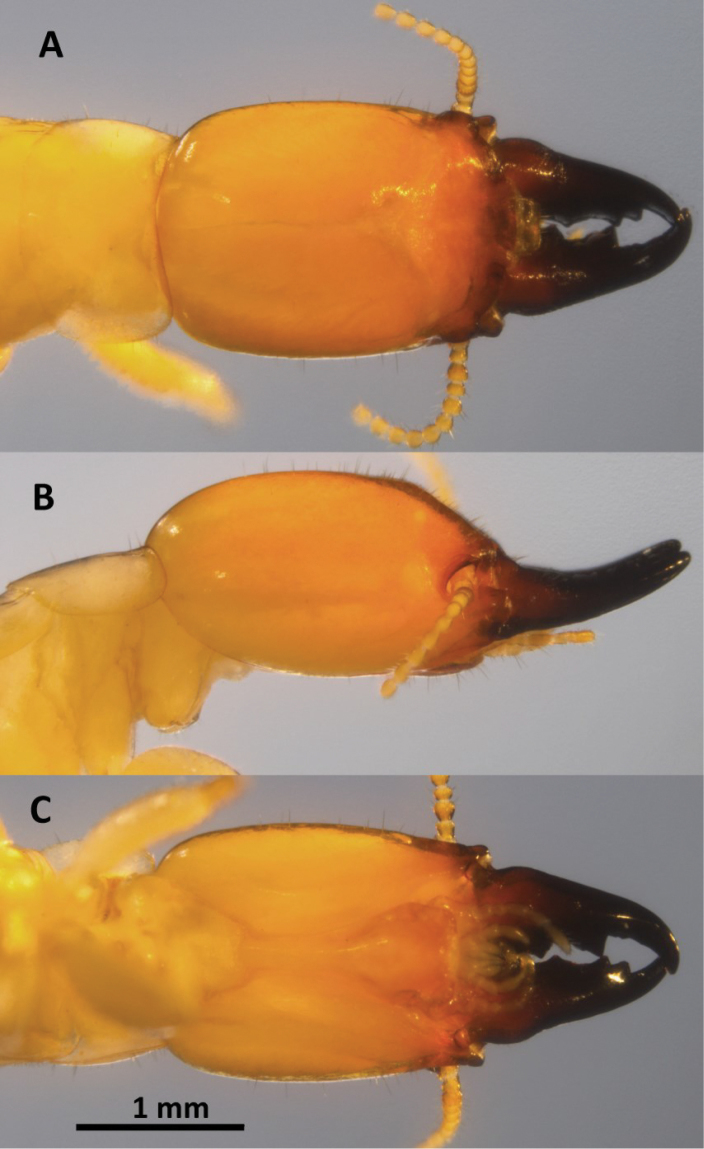
Head and pronotum of *Neotermes agilis* soldier**. A** Dorsal **B** lateral, and **C** ventral views.

*Soldier* (Fig. 6; Table 4). Monomorphic. Head capsule dark redish brown in a narrow strip anterior to antennae; remainder of head capsule light yellowish orange. Mandibles black beyond humps, humps dark reddish brown. Eye spots faint or absent. Pronotum light yellowish orange. Head capsule in dorsal view, subquadrate; lateral margins slightly convex. In lateral view, head capsule ellipsoid. Frons sloping from vertex ≅40°; mandibles curved upward ≅20°. Scattered medium and short setae on head capsule and pronotum. Periantennal carina weak; mandibles short and stout; left mandible with three submarginal teeth, right mandible with two large subapical teeth, distal tooth forming right angle with apical tooth, apical teeth angled 50°; basal humps slightly inflated. Labrum linguiform, medium-sized, with long terminal setae. Antennae with 12 articles, third article somewhat enlarged but shorter than fouth and fifth combined. Pronotum as broad as head, wide; anterior margin shallowly incised; anterolateral corners rounded ≅70°, lateral margins parallel; posterior margin rectate in middle. Front femora inflated.

**Table 4.** Measurements of *Neotermes agilis* soldier (n=2).

Measurement	max.	min.	mean
Head length to tip of mandibles	3.30	3.20	3.25
Head length to postclypeus	2.20	2.20	2.20
Head width, maximum	1.55	1.50	1.53
Span of frontal processes	0.95	0.80	0.88
Head height, excluding postmentum	1.25	1.25	1.25
Labrum, maximum width	0.38	0.25	0.31
Postclypeus width, maximum	0.43	0.40	0.41
Left mandible length, tip to most distant visible point of ventral condyle	1.25	1.20	1.23
Postmentum, length in middle	1.35	1.20	1.28
Postmentum, maximum width	0.63	0.48	0.55
Postmentum, minimum width	0.18	0.15	0.16
Pronotum, maximum width	1.50	1.45	1.48
Pronotum, maximum length	0.95	0.85	0.90
Hind tibia length	0.83	0.83	0.83
Total length	7.78	7.62	7.70
No. antennal articles	12	12	12

#### Comparisons.

The fore wing venation of *N. agilis* is unique among *Neotermes* and other kalotermitids in that the major veins are hyaline beyond their bases and the radial sector and median veins coalesce to form a single vein. The venation of *N. agilis* is close to *Rugitermes*, but in *Rugitermes* the distal two thirds of median vein is sclerotized. All other *Neotermes* have fore wings with fully sclerotized major veins and a median vein which remains independent of the radial sector (see Ware et al. 2010). The soldier of *N. agilis* is among the smallest of the genus and has an unusually broad pronotum.

## Discussion

As with many older works, Sjöstedt’s (1925) description of the soldier of *N. aburiensis* is brief and lacks illustrations. However, he describes the specimen collected “from old wounds in stems of cocoa tree” in Aburi, Ghana, as having its “forehead depressed in back; bordered by a curved keel” (translated from German) and having a head width of 2–2.3 mm. Grassé (1937) described the winged imago of *N. aburiensis* from a cacao trunk in Bingerville, Côte d’Ivoire. He included a detailed drawing of the fore wing (11 mm long) and sketches of the soldier (with curved frons and lateral protuberances), nymph, and soldier antennae. Coaton’s 1955 lateral drawing of the *N. aburiensis* soldier head capsule hints of a frontal cleft, and he notes that the “frons is depressed medially, bordered behind by a distinct, concavely curved ridge”. Coaton (1955) examined four soldiers from an extreme easterly locality at Lake Albert, Congo.

Sjöstedt’s (1904) description, including measurements, of the *N. agilis* imago from Johann Albrechtshöhe station, Cameroon, was compared to our specimens from Ghana. Although lacking illustrations, the body morphology reported by Sjöstedt (1904) matches closely with our material. He notes “wings thin, narrow with constricting rounded tips......the basal third of the costal and median and the inner branch of the radial sector yellow brown or yellow red..... the remainder is hyaline” (translated from German). The partial pigmentation of the wings is used in Sjöstedt’s (1925) key for African *Neotermes*. When RHS first examined the imago of *N. agilis*, the venation did not support inclusion in *Neotermes*. When the imago and soldier specimens were examined by Kumar Krishna in 2006, he placed the specimens in *Neotermes* without comment on morphology. With great respect for Dr. Krishna’s knowledge of the group, we keep *N. agilis* in *Neotermes* for now.

## Supplementary Material

XML Treatment for Neotermes aburiensis

XML Treatment for Neotermes agilis
